# Loss of Rab44 attenuates ovalbumin-induced allergic airway inflammation by modulating immune responses and eosinophil function

**DOI:** 10.3389/fimmu.2026.1820891

**Published:** 2026-05-21

**Authors:** Haruka Mawatari, Yu Yamaguchi, Takao Ayuse, Takuro Sanuki, Tomoko Kadowaki, Takayuki Tsukuba

**Affiliations:** 1 Department of Dental Pharmacology, Graduate School of Biomedical Sciences, Nagasaki University, Nagasaki, Japan; 2 Department of Clinical Physiology, Graduate School of Biomedical Sciences, Nagasaki University, Nagasaki, Japan; 3 Clinical Research Center, Nagasaki University Hospital, Nagasaki, Japan; 4 Department of Frontier Oral Science, Graduate School of Biomedical Sciences, Nagasaki University, Nagasaki, Japan

**Keywords:** allergic airway inflammation, eosinophils, immune responses, ovalbumin, Rab44

## Abstract

**Introduction:**

Asthma is a chronic inflammatory disease of the airways accompanied by mucus hypersecretion, airway remodeling, and bronchial hyperresponsiveness. However, the mechanisms by which membrane trafficking molecules contribute to the development of asthma remain unclear. In the present study, we investigated the role of Rab44 in a murine model of allergic airway inflammation mimicking human asthma, as we previously identified Rab44 and are exploring its function.

**Methods:**

Experimental allergic airway inflammation was induced in Rab44-knockout (KO) and wild type (WT) mice sensitized to ovalbumin (OVA). Histopathological analysis, RNA sequencing (RNA-seq) analysis, degranulation assays, adhesion and migration assays, and flow cytometric analysis of adhesion and chemokine receptors were performed.

**Results:**

Compared with WT mice, Rab44-KO mice exhibited impaired OVA-induced allergic airway inflammation. RNA-seq analysis revealed that Rab44 deficiency affected several signaling pathways involved in immune and inflammatory responses in the lungs of OVA-induced mice. Moreover, the mRNA levels of cytokines and the numbers of lymphocytes, monocytes, and eosinophils infiltrating the bronchoalveolar lavage fluid were decreased in KO mice compared with WT mice. In cultures of eosinophils derived from bone marrow cells, Rab44-KO eosinophils exhibited aberrant differentiation and impaired release of eosinophil peroxidase but not major basic protein. Rab44-KO eosinophils exhibited reduced cell adhesion and chemotaxis. Consistent with these findings, Rab44-KO eosinophils showed impaired surface expression of adhesion and chemokine receptors.

**Conclusion:**

These results indicate that Rab44 deficiency attenuates OVA-induced allergic airway inflammation by modulating immune responses and eosinophil function.

## 1 Introduction

Asthma is a chronic inflammatory disease of the airways characterized by mucus hypersecretion, airway remodeling, and bronchial hyperresponsiveness (1). Although multiple asthma phenotypes exist, T2-high type asthma is the most representative phenotype and is marked by eosinophil infiltration from the blood into the inflamed lungs and airways and secretion of Th2-type mediators, such as interleukin (IL)-4, IL-5, IL-13, IgE, leukotrienes, and prostaglandins (2). Among Th2 type-mediated immune cells, eosinophils play a central role in T2-high asthma. Upon inflammatory stimulation, eosinophils degranulate to release several cationic proteins such as major basic protein (MBP) and eosinophil peroxidase (EPX), which are stored within cytoplasmic granules (3, 4). Because the pathophysiology of asthma is highly complex, animal models are useful for studying the molecular mechanisms underlying the pathological conditions of human asthma (5, 6). Ovalbumin (OVA) is commonly used as an antigen in experimental asthma models. Both OVA and Th2-adjuvant aluminum hydroxide are required to sensitize the mice to OVA-induced inflammation (7). Therefore, we investigated the role of Rab44 in allergic airway inflammation using an experimental mouse model.

Rab GTPases are the key regulators of membrane trafficking in specific cell types, including immune-related cells, osteoclasts, and myoblasts (8–10). Rab44 is classified as a large Rab GTPase existing two isoforms with molecular weights of approximately 75–100 kDa that possesses additional domains, such as the EF-hand, coiled-coil, proline-rich, and Rab domains (11, 12). Therefore, Rab44 differs from conventional small Rab GTPases, including Rab1-43, which have low molecular weights of approximately 20–30 kDa (12, 13). Several lines of evidence indicate that Rab44 is responsible for the differentiation of osteoclasts and myoblasts (11, 14, 15). Moreover, Rab44 is highly expressed in immune-related cells and is most abundant in the granulocyte and mast cell lineages (16, 17). We previously reported that Rab44-knockout (Rab44-KO) mice show decreased anaphylactic responses and reduced nickel-induced hypersensitivity (18–20). Furthermore, histamine and lysosomal enzymes released from the bone marrow-derived mast cells of Rab44-KO mice were lower than those released from wild type (WT) mice (17, 18). Considering that Rab44 is involved in the membrane trafficking and differentiation of immune cells, we speculated that Rab44 regulates allergic asthma. However, it remains unclear whether Rab44 deficiency induces abnormal phenotypes in mice and eosinophils during OVA-induced allergic airway inflammation.

In this study, we induced allergic airway inflammation in Rab44-KO mice using OVA to examine the effects of Rab44 on inflammation and cell infiltration and compared the functions of eosinophils derived from WT and Rab44-KO mice.

## 2 Materials and methods

### 2.1 Antibodies and reagents

Fluorescein isothiocyanate (FITC) anti-mouse/rat CD29 (integrin β1) antibody (cat. no. BL102205), FITC anti-mouse CD49d (α4 integrin antibody; cat. no. BL103605), FITC anti-mouse CD44 antibody (cat. no. BL156007), FITC anti-mouse CD193 (CCR3) (cat. no. BL144509), FITC anti-mouse CD191 (CCR1) antibody (cat. no. BL152505), FITC Armenian Hamster IgG isotype control antibody (cat. no. BL400905), FITC rat IgG2a, κ isotype control antibody (cat. no. BL400505), FITC rat IgG2b, and κ isotype control antibody (cat. no. BL400605) were purchased from BioLegend (London, UK). Anti-mouse FcR blocking reagent (cat. no. 130-092-575) was obtained from Miltenyi Biotec. Aluminum hydroxide gel adjuvant (cat No. LSL/LG-6000) was purchased from LSL/Cosmo Bio (Tokyo, Japan).

### 2.2 Animals

Rab44-KO mice were generated from a C57BL/6 background strain using CRISPR/Cas9-mediated genome editing, as described previously (18). *In vivo* animal experiments were performed using age-matched (8–14 weeks old) female WT and Rab44-KO littermates. The number of mice used is indicated as “n” in the respective figure legends. The mice were euthanized by gradual-fill carbon dioxide (CO_2_) inhalation, and death was confirmed by cervical dislocation. All the animal experimental protocols were approved by the Animal Experiment Committee of the Graduate School of Biomedical Sciences, Nagasaki University (permit number: 2107211733).

### 2.3 Allergen-induced pulmonary inflammation

Mice were sensitized via intraperitoneal injection of 100 μg of ovalbumin (OVA; Sigma-Aldrich, St. Louis, MO, USA) emulsified in aluminum hydroxide gel adjuvant on days 0, 7, and 14. From days 17 to 20, mice were anesthetized and placed in the supine position with their limbs immobilized. OVA (100 μg) dissolved in 50 μL of endotoxin-free phosphate-buffered saline (PBS) was administered intranasally using a micropipette in 2 μL aliquots per administration. Intranasal administration was performed according to the method described by Suzuki et al. (21) with some modifications. Bronchoalveolar lavage fluid (BALF) and lung tissue samples were collected on day 21. BALF was collected as previously described by Mo et al. (22) with some modifications. Briefly, the mice were anesthetized with a mixture of midazolam (4 mg/kg), butorphanol tartrate (5 mg/kg), and hydrochloric acid medetomidine (0.3 mg/kg) by intraperitoneal administration, placed in the supine position, and subjected to tracheotomy. A tracheal cannula (3 Fr Atom Pink catheter) was inserted into the trachea, and 0.5 mL of Ca^2+^- and Mg^2+^-free PBS containing 0.1% bovine serum albumin (BSA) and 0.05 mM EDTA-2Na was instilled into the airway and gently aspirated. This lavage procedure was repeated four times per cycle, and three cycles were performed for a total of 12 repetitions to collect BALF.

### 2.4 Histopathology

Lung sections were harvested from the OVA-induced mice. The specimens were fixed in 4% paraformaldehyde and processed to prepare paraffin-embedded sections. Sections were stained with hematoxylin and Periodic acid-Schiff (PAS) to assess mucin production. PAS-positive cells are expressed as the percentage of PAS-positive epithelial cells relative to total epithelial cells. In addition, quantification was performed in a blind manner across multiple bronchioles per mouse (total 10 airways).

### 2.5 Measurement of cytokine levels using enzyme-linked immunosorbent assay

Levels of cytokines, including IL-5, IL-13, IL-17A, and interferon (IFN)-γ in the BALF from OVA-sensitized mice were quantified using commercially available ELISA kits (R & D Systems, Minneapolis, MN, USA).

### 2.6 RNA-sequencing and data analysis

Total RNA was isolated from the lungs of normal, OVA-administered WT and KO mice. RNA was extracted using the NEBNext Ultra II Directional RNA Library Prep Kit (New England Biolabs) according to the manufacturer’s instructions. RNA sequencing and subsequent bioinformatic analyses were performed by Rhelixa Co., Ltd. (Tokyo, Japan). Briefly, the quality and quantity of the RNA were assessed using an Agilent 2100 Bioanalyzer (Agilent Technologies) and a NanoDrop spectrophotometer (Thermo Fisher Scientific). RNA-seq libraries were prepared following the manufacturer’s protocol and sequenced on an Illumina NovaSeq X Plus platform to generate 150-bp paired-end reads. Raw sequencing reads were subjected to quality control and trimming, and the processed reads were aligned to the mouse reference genome (GRCm38) using the HISAT2 software. The gene expression levels were quantified using featureCounts software. Differentially expressed gene (DEG) analysis was performed using DESeq2, and genes with an adjusted p-value < 0.05 were considered significantly differentially expressed. Functional enrichment analyses, including Gene Ontology (GO) and Kyoto Encyclopedia of Genes and Genomes (KEGG) pathway analyses, were conducted using the Cluster Profiler R package (version 4.10.0).

### 2.7 Culture of bone marrow-derived eosinophils

Differentiation of eosinophils from the bone marrow cells of 12–24-week-old and sex-matched mice was performed according to the method described by Stoeckle et al. (23), with some modifications. Isolated cells were incubated at 37 °C in an atmosphere of 5% CO_2_ in Iscove’s modified Dulbecco’s medium (IMDM) containing 10% fetal bovine serum, 2 mM L-glutamine, 5 × 10^-5^ M 2-mercaptoethanol, 1% penicillin, and streptomycin in the presence of 100 ng/mL Flt3L and 100 ng/mL SCF (Peprotech) for 4 days, followed by 10 ng/mL IL-5 (R&D Systems) for 7 days, with medium replacement every 3 days. Eosinophils were stained with Wright-Giemsa stain and counted under an optical microscope.

### 2.8 Reverse transcription quantitative polymerase chain reaction

Reverse transcription quantitative polymerase chain reaction (RT-qPCR) analysis was performed as described previously (11). Briefly, total RNA was extracted from the bone marrow-derived eosinophil cells using TRI reagent (Molecular Research Center Inc. Cincinnati, OH, USA). Reverse transcription was performed using oligo(dT) 15 primer (Promega, Tokyo, Japan) and ReverTra Ace (Toyobo, Osaka, Japan). RT-qPCR was conducted using a Quantstudio3 system (Applied Biosystems, Waltham, MA, USA). The cDNA was amplified using Brilliant III Ultra-Fast SYBR Green QPCR Master Mix (Agilent Technologies, Hachioji, Tokyo, Japan). The following primer sets were utilized. *Gapdh*, forward: AAATGGTGAAGGTCGGTGTG and reverse: TGAAGGGGTCGTTGATGG; *IL-5Rα*, forward: CAGTGGGAGAAACCACTTTCTGCC and reverse: GAGATGCCATTCTACCAAGGACTTC; *Ccr3*, forward: TCA ACT TGG CAA TTT CTG ACC T and reverse: CAG CAT GGA CGA TAG CCA GG; *CD11b*, forward: TACTTCGGGCAGTCTCTGAGTG and reverse: ATGGTTGCCTCCAGTCTCAGCA; *Ly6G*, forward: AGA AGC AAA GTC AAG AGC AAT CTC T and reverse: TGA CAG CAT TAC CAG TGA TCT CAG T; *CD62L*, forward: AATGGCCGTGGAGAATGTGT and reverse: CTGGACCACTTGGCAGATTG.

### 2.9 Degranulation assay

Cultured eosinophils (1 × 10^5^ cells/well) were stimulated at 37 °C for 2 h with 100 ng/mL eotaxin, 100 ng/mL RANTES, or 5 μg/mL A23187 in complete IMDM. The samples were centrifuged at 52.5 × *g*, at 4 °C for 10 min. The supernatants were collected to measure the amounts of MBP and EPX released. For degranulation assays, 100 µL of the cell culture supernatant was used according to the method of mouse MBP ELISA kit (Bioss antibodies Inc. No: BSKM62441) or a mouse EPX ELISA kit (CUSABIO Inc. CSB E15830m).

### 2.10 Cell adhesion assay

Plastic 96-well plates were coated with PBS or fibronectin (25 μg/mL) in sterile distilled water and incubated for 1 h at 25 °C. The wells were blocked with IMDM containing 0.5% BSA. After washing with IMDM containing 0.1% BSA (washing buffer), cultured eosinophils (1 × 10^5^ cells) were added to each well and incubated at 37 °C for 60 and 120 min. After gentle washing with washing buffer, the bound cells were fixed with 4% paraformaldehyde in PBS and stained with 50 µL of Coomassie Brilliant Blue (5 mg/mL in 2% ethanol). After washing with the washing buffer, the number of stained cells per unit area was counted under a light microscope.

### 2.11 Chemotaxis assay

Chemotaxis assays were performed as previously described with some modifications (24). Cultured eosinophils (5 × 10^5^ cells/50 µL) were washed and resuspended in IMDM. Migration was assessed in a 24-well chemotaxis chamber (Transwell^®^, Corning^®^) separated by a polyethylene terephthalate membrane (pore size, 5 μm). Eosinophils were loaded into the upper chambers and tested for chemoattraction to IMDM medium with 10% fetal bovine serum or 200 ng/mL RANTES. The chambers were incubated at 37 °C in 5% CO_2_ for 60 or 120 min. Cells that migrated to the lower wells were fixed with 4% paraformaldehyde in PBS for 10 min, stained with Coomassie Brilliant Blue (5 mg/mL in 2% ethanol), and counted under a light microscope.

### 2.12 Cell staining and flow cytometric analysis

Cultured eosinophil suspensions (2 × 10^5^ cells/100 µL) were incubated on ice for 15 min with primary antibodies diluted in PBS containing 2% FBS (buffer A). The samples were preincubated with an FcR-blocking reagent and subsequently with specific antibodies or control antibodies conjugated with FITC for 15 min on ice. After washing three times with buffer A, flow cytometry analysis was performed using a BD FACSVerse™ cytometer (BD Bioscience).

### 2.13 Statistical analysis

Quantitative data are presented as mean ± standard deviation (SD). For non-parametric analyses, where a normal distribution cannot be assumed due to the small sample size, the Mann-Whitney U test was used. For a multi-group analysis, the Tukey-Kramer method was used to identify differences between each experimental group when a significant difference (*p < 0.05 or **p < 0.01) was determined using analysis of variance (ANOVA).

## 3 Results

### 3.1 Rab44 deficiency impairs OVA-induced allergic airway inflammation

**Figure 1:**
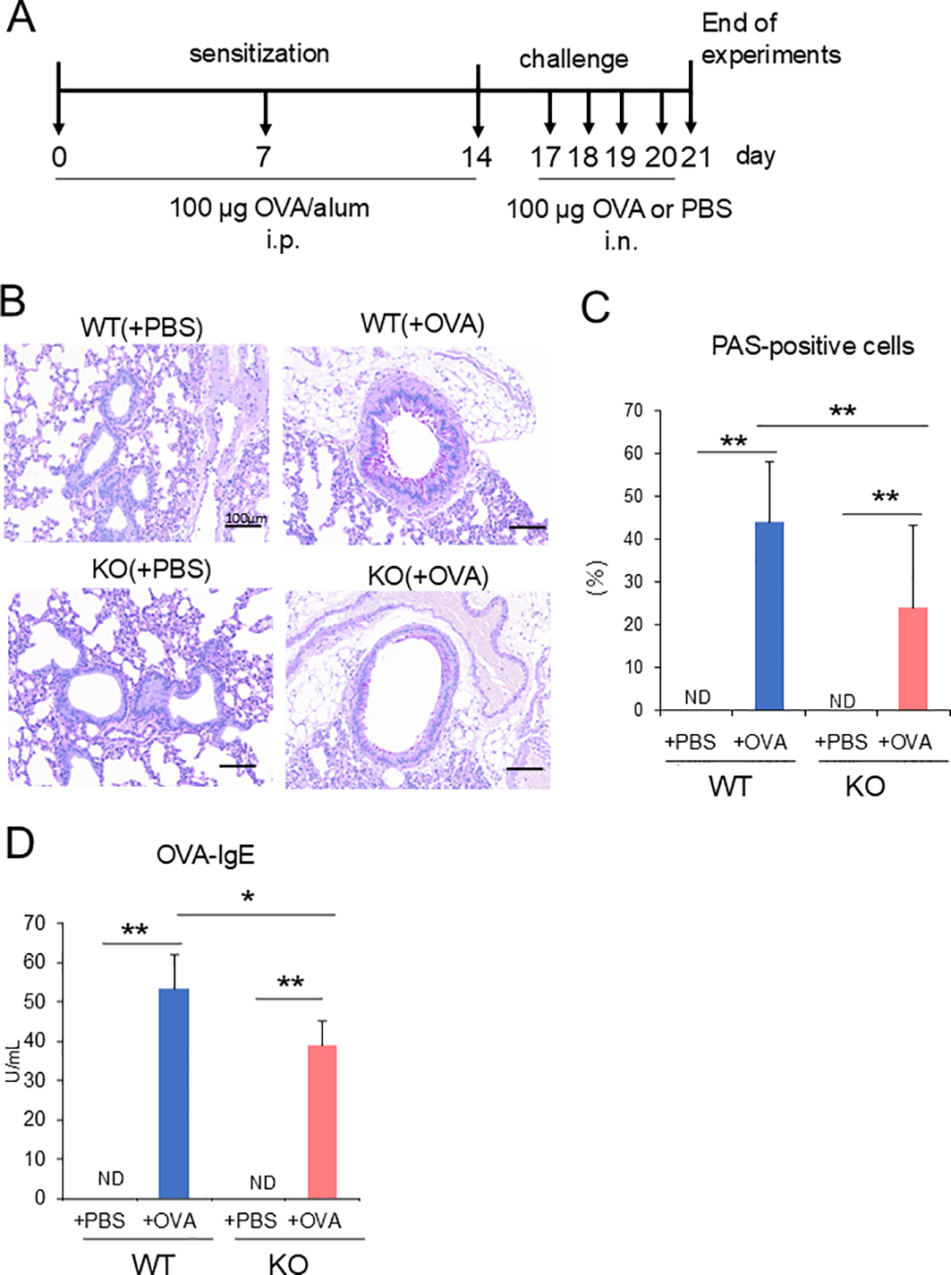
Development of ovalbumin (OVA)-induced allergic asthma in mice. Wild type (WT) and Rab44-knockout (KO) mice were intraperitoneally injected with ovalbumin (OVA) and aluminum hydroxide (alum) (100 μg) on days 0, 7, and 14; subsequently they were intranasally challenged with 50 μL OVA (100 μg) or 50 μL PBS for 4 days. **(A)** A schematic diagram showing the time course of the sensitization protocol for OVA-induced allergic asthma. **(B)** Representative images of histological analysis using Periodic acid-Schiff (PAS) staining of lung sections from OVA-treated mice on day 21. **(C)** PAS-positive scores in OVA-treated WT and Rab44-KO mice. Scores were calculated as described in the Materials and Methods section. WT: n = 10; Rab44-KO: n = 10 (10 airways of 5 mice) *p < 0.05, **p < 0.01. The Tukey-Kramer method was used.

To determine whether Rab44 is implicated in the development of allergic airway inflammation sensitized with OVA, we performed *in vivo* experiments in WT and Rab44-KO mice. **Figure 1A** shows the time course of the OVA-induced allergic airway inflammation experiments. Histopathological analysis of the lung sections using PAS staining revealed an increased inflammatory reaction in the peribronchial and perivascular areas, which were surrounded by infiltrating inflammatory cells in WT mice compared with those in Rab44-KO mice under OVA-induced conditions (**Figure 1B**). However, no significant difference was observed between WT and Rab44-KO mice following PBS treatment (**Figure 1B**). Quantification of PAS-positive cells showed that the number of PAS-positive cells in Rab44-KO mice was significantly lower than that in WT mice under OVA-induced conditions (**Figure 1C**). These results indicate that Rab44 deficiency impairs OVA-induced allergic airway inflammation in mice.

### 3.2 Rab44 deficiency affects several signaling pathways involved in immune and inflammatory responses in the lung of OVA-induced mice

**Figure 2:**
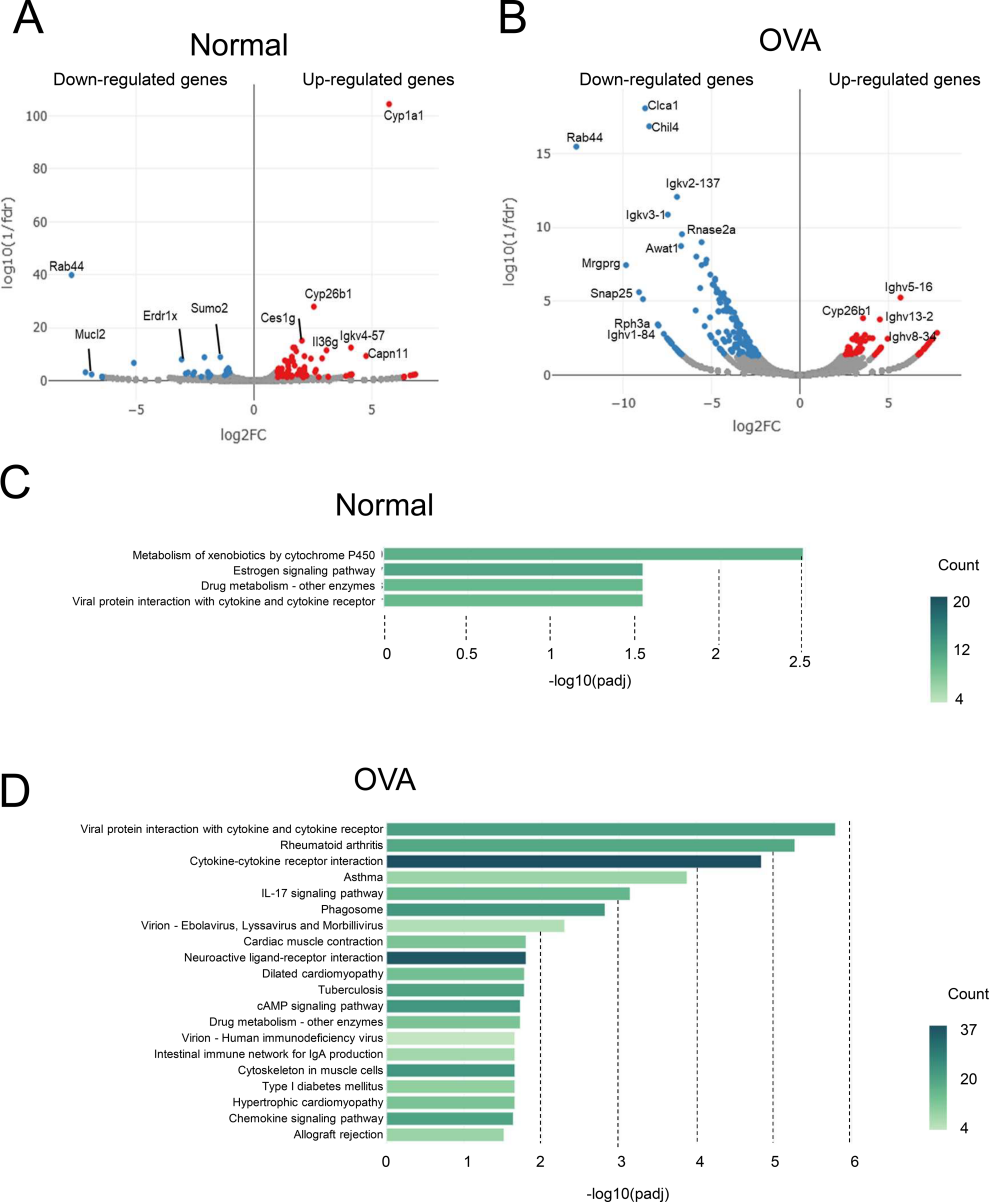
RNA-seq analysis of altered genes and pathways in the lungs of normal and OVA-induced mice. Total RNA was isolated from the lungs of normal and OVA-induced WT and KO mice. Sequencing data of the extracted RNA was analyzed as described in the “Materials and Methods”. Volcano plots of differentially expressed genes (DEGs) (WT vs. KO). Dysregulated genes are defined by the absolute value of the log2 (ratio to WT) with p-value. The cutoff criteria for differentially expressed genes. The criteria is |Expression variation ratio| > 1 and adjusted P-value < 0.05. Red: Up-expression genes, Blue: Down-expression genes. Gray: Other genes. **(A)** Normal conditions; **(B)** OVA-induced conditions. **(C, D)** Kyoto Encyclopedia of Genes and Genomes (KEGG) pathway enrichment analyses of the lungs of normal and OVA-induced WT and KO mice. **(C)** Normal conditions. **(D)** OVA-induced conditions.

To investigate whether Rab44 deficiency affects gene expression and signaling pathways, we analyzed global gene expression in the lungs of WT and Rab44-KO mice under normal and OVA-induced conditions using RNA-seq (**Figure 2**). Under normal conditions, genes encoding cytochrome P450 (CYP) enzymes, such as *Cyp1a1* and *Cyp26b1*, were upregulated in the lungs of Rab44-KO mice compared with those in WT mice (**Figure 2A**, **Supplementary Table 1**). Interestingly, under OVA-induced conditions, chloride channel accessory 1 (*Clca1*), which regulates chloride ion channels and mucus production in the airway epithelium, and chitinase-like 4 (*Chil4*), also known as *Ym2*, which is a marker of Th2-type and eosinophilic inflammation, were markedly downregulated in the lungs of Rab44-KO mice (**Figure 2B**, **Supplementary Table 2**). Moreover, several immunoglobulin-related genes, such as immunoglobulin heavy chain variable (*ighv*) and immunoglobulin kappa variable (*igkv*), were upregulated and downregulated, respectively, in the lungs of Rab44-KO mice compared with those in WT mice (**Figure 2B**, **Supplementary Table 2**). GO and KEGG functional enrichment analyses of the DEGs revealed that several pathways involved in immune and inflammatory responses were altered under OVA-induced conditions (**Figure 2D**, **Supplementary Table 4**), whereas some metabolism-related pathways were affected under normal conditions (**Figure 2C**, **Supplementary Table 3**). Notably, under OVA-induced conditions, the top five enriched signaling pathways detected were viral protein interactions with cytokines and cytokine receptors, rheumatoid arthritis, cytokine-cytokine receptor interactions, asthma, and the IL-17 signaling pathway (**Figure 2D**, **Supplementary Table 4**). Thus, Rab44 deficiency affects several signaling pathways involved in the immune and inflammatory responses under OVA-induced conditions.

### 3.3 Rab44 deficiency attenuates immune responses in BALF in OVA-induced mice

**Figure 3:**
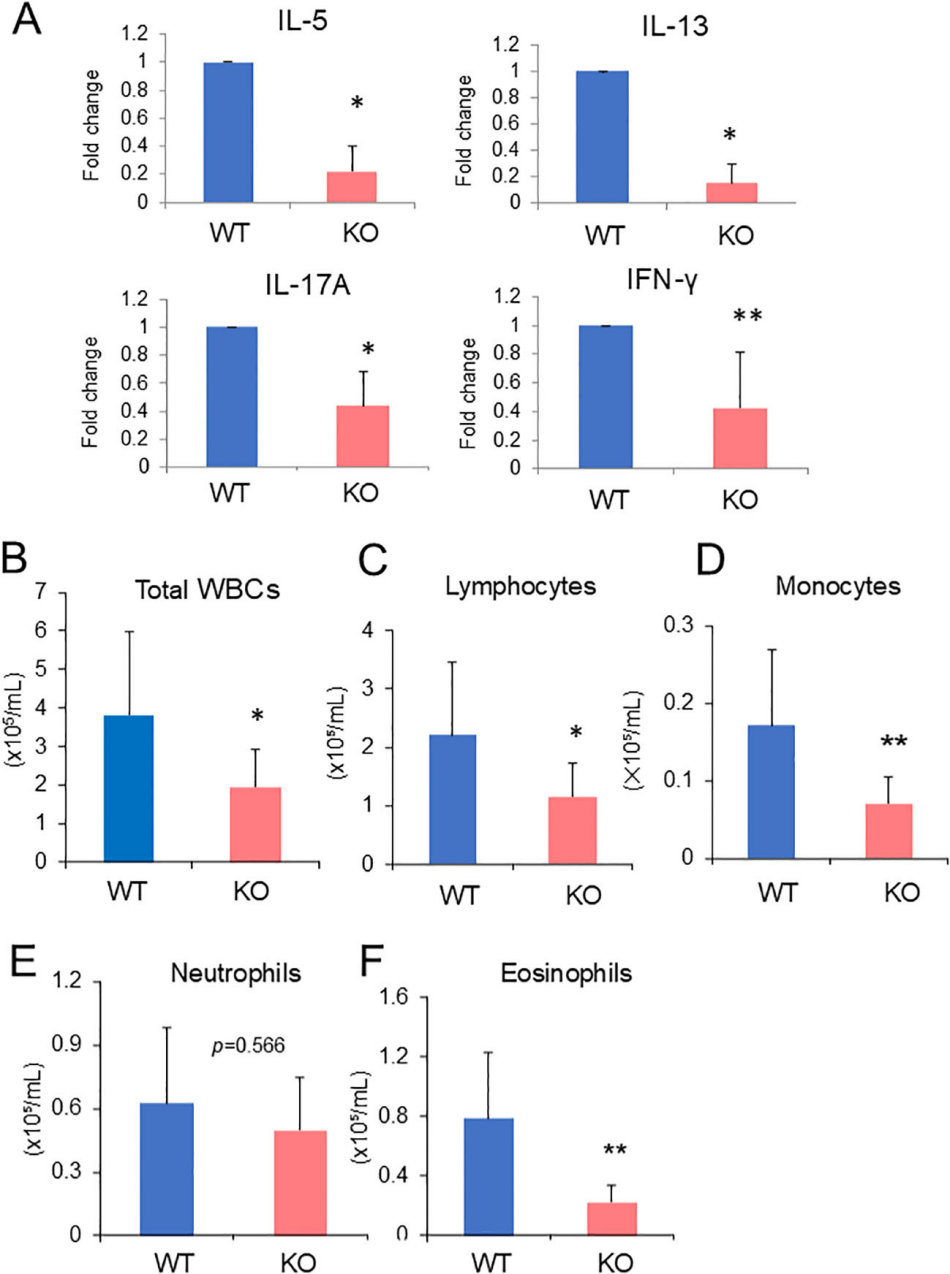
Immunological and cell biological analyses of the BALF of OVA-induced mice. **(A)** Cytokine production levels in the BALF of OVA-induced WT and Rab44-KO mice. Interleukin (IL)-5, IL-13, IL-17A, and interferon (IFN)-γ. **(B–D)** The numbers of total white blood cells (WBCs), lymphocytes, monocytes in the BALF of WT and Rab44-KO mice after OVA-induction using a hematology counter Thika (ARKRAY). WT, n = 8; KO, n =8 (8 mouse samples). **(E, F)** The numbers of neutrophils and eosinophils were identified in the BALF smears stained using Wright-Giemsa staining under a light microscope. n = 8; KO, n =8. (8 mouse samples) *p < 0.05, **p < 0.01. The Mann-Whitney U test was used.

We assessed cytokine production in the BALF of WT and Rab44-KO mice following OVA-induction. The levels of the Th2 cytokines, IL-5 and IL-13, produced in the BALF of Rab44-KO mice were significantly lower than those in WT mice following OVA-induction (**Figure 3A**). Moreover, the levels of IL-17A, a marker of Th17 responses, and IFN-γ, a marker of Th1 responses, in the BALF of Rab44-KO mice were also significantly lower in that of WT mice following OVA-induction (**Figure 3A**). Thus, Rab44 deficiency reduces a wide range of immune responses in BALF.

We further analyzed the profiles of white blood cell types in the BALF of WT and Rab44-KO mice under OVA-induced conditions using a hematology counter and Wright-Giemsa staining. The white blood cell count in the BALF of Rab44-KO mice was significantly lower than in WT mice (**Figure 3B**). Moreover, the numbers of lymphocytes, monocytes, and eosinophils in Rab44-KO mice were significantly lower than those in WT mice (**Figures 3C, D, F**). However, the number of neutrophils did not significantly differ in the BALF of WT and Rab44-KO BALF samples (**Figure 3E**). These results indicate that Rab44 deficiency affects the infiltration of white blood cells into the BALF of mice. In particular, eosinophil infiltration into BALF of Rab44-KO mice was markedly impaired.

### 3.4 Rab44-KO eosinophils display aberrant differentiation

**Figure 4:**
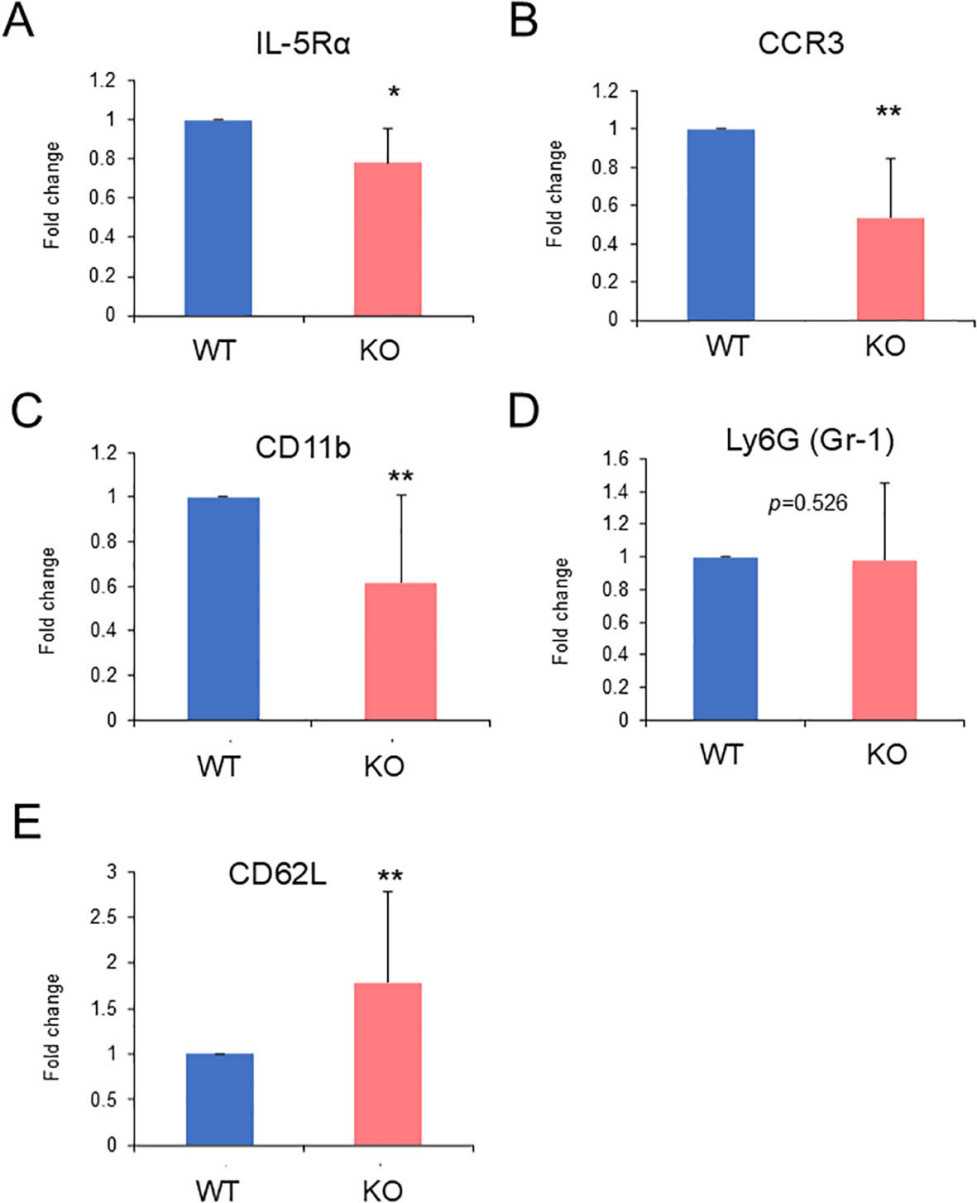
Comparison of mRNA levels of various eosinophils marker molecules in bone marrow cells. Bone marrow cells were cultured with Dulbecco’s medium (IMDM) containing 10% fetal bovine serum in the presence of 100 ng/mL Flt3L and 100 ng/mL SCF for 4 days and subsequently added with 10 ng/mL IL-5 for 7 days. After mRNA isolation from these cells, RT-PCR was performed. **P < 0.01, compared with the WT cells. The data are represented as mean ± SD of values from more than six independent experiments. **(A)** IL-5Rα, **(B)** CCR3, **(C)** CD11b, **(D)** Ly6G, and **(E)** CD62L. *p < 0.05, **P < 0.01; compared with WT cells. The Mann-Whitney U test was used.

Next, we performed *in vitro* experiments using eosinophils cultured from bone-marrow cells of WT and Rab44-KO mice. When bone-marrow cells derived from WT and KO mice were cultured for 11 days, we found that eosinophil purity, assessed by Wright-Giemsa staining, was over 90% for both WT and Rab44-KO cells, with no morphological abnormalities (data not shown). To further examine differentiation into eosinophils, we analyzed the mRNA levels of several eosinophil marker genes in WT and Rab44-KO cells. The mRNA levels of IL-5Rα, CCR3 (mature eosinophil markers), and CD11b (a myeloid cell marker), were significantly lower than those in WT cells (**Figures 4A–C**). The mRNA levels of Ly6G (a granulocyte marker) between WT and Rab44-KO cells were similar (**Figure 4D**). However, the mRNA levels of CD62L (a marker for eosinophil subpopulations), were significantly higher than those in WT cells (**Figure 4E**). These results indicate that Rab44-deficient bone-marrow cells exhibit aberrant differentiation into eosinophils.

### 3.5 Rab44-KO eosinophils exhibit impaired degranulation of EPX but not MBP

**Figure 5:**
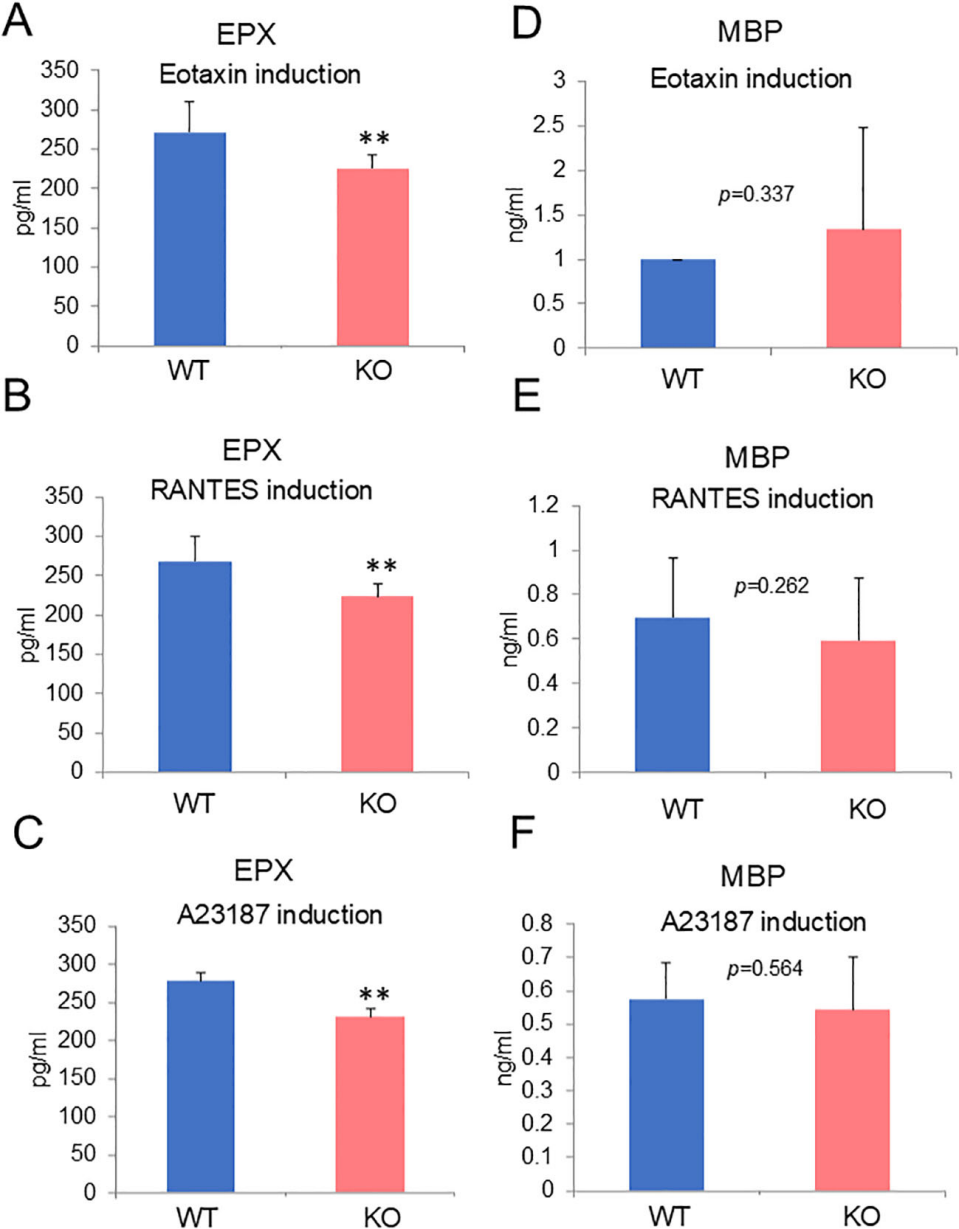
Secretion levels of EPX and MBP from bone marrow eosinophils. Bone marrow eosinophils (5 × 10^5^ cells) were seeded on plastic wells and subsequently stimulated with eotaxin (100 ng/mL), RANTES (100 ng/mL), or A23187, a Ca^2+^ ionophore, (5 μg/mL), and further incubated at 37 °C for 2 h. The collected samples were centrifuged at 52.5 × *g*, 4 °C, for 10 min. EPX or MBP levels in the supernatants were analyzed using ELISA. Data are represented as the mean ± S.D. from four independent experiments. **(A–C)** EPX, **(D–F)** MBP, **(A, D)** Eotaxin stimulation; WT: n = 8; Rab44-KO: n = 8 (samples of eosinophils).**p < 0.01; **(B, E)** RANTES stimulation. WT: n = 8; Rab44-KO: n =8 (samples of eosinophils). **p < 0.01; **(C)** and **(F)** A23187 stimulation WT: n = 6; Rab44-KO: n = 6 (samples of eosinophils), **p < 0.01. The Mann-Whitney U test was used.

To investigate the effects of Rab44 on eosinophil degranulation, we measured the levels of EPX and MBP released. Eotaxin and RANTES are chemokine ligands that promote the degranulation of intracellular granules in eosinophils (25). In addition, the A23187 calcium ionophore, which is triggered by Ca^2+^ influx, promotes the secretion of granule contents (26). When WT and Rab44-KO eosinophils were stimulated with eotaxin, RANTES, or A23187, the secretion levels of EPX in Rab44-KO eosinophils were significantly lower than those in WT cells (**Figures 5A–C**). In contrast, the secretion levels of MBP from the secretory granules in WT and Rab44-KO eosinophils were similar (**Figures 5D–F**). These results indicate that Rab44-KO eosinophils exhibit impaired degranulation of EPX, but not MBP.

### 3.6 Rab44-KO eosinophils exhibit reduced cell adhesion ability and decreased surface levels of adhesion receptors

**Figure 6:**
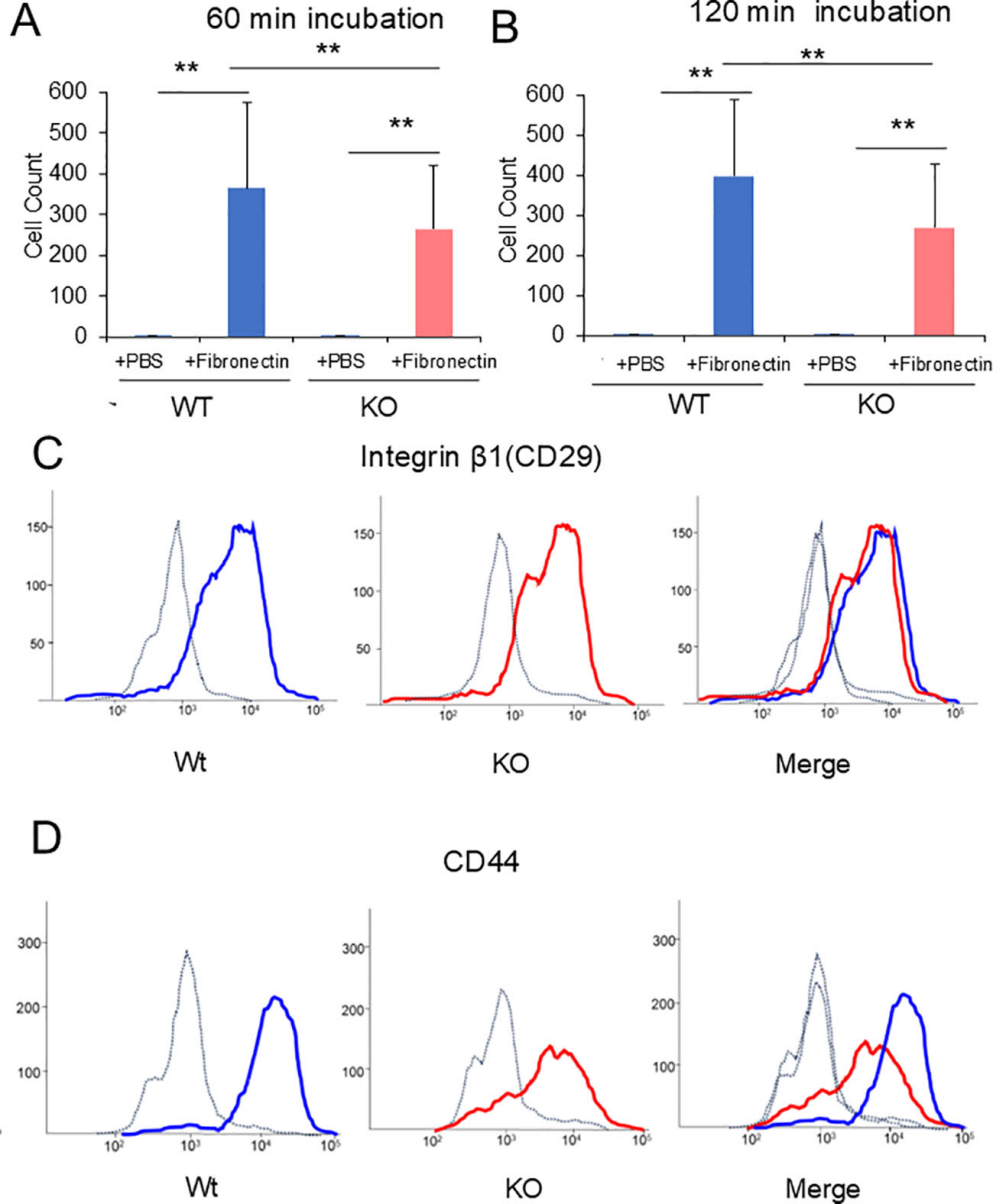
Cell adhesion and surface levels of adhesion receptors in bone marrow eosinophils. **(A, B)** Bone marrow eosinophils (1 × 10^5^ cells) were added to fibronectin-treated plastic wells and subsequently incubated at 37 °C for 60 **(A)** or 120 min **(B)**. After washing, the bound cells were fixed with 4% paraformaldehyde and subsequently stained with 5% Coomassie Brilliant Blue. The number of stained cells per the same area were counted using a light microscope. WT: n = 5; Rab44-KO: n = 5 (samples of eosinophils); **p < 0.01. Data are representative of six independent experiments. **(C, D)** The surface of the bone-marrow eosinophils (1 × 10^5^ cells) was stained with FITC-labeled antibodies for integrin β1(CD29) **(C)** or CD44 **(D)** and then analyzed using flow cytometry. Data are representative of five independent experiments. The Tukey-Kramer method was used.

To investigate whether Rab44 is involved in receptor expression, we analyzed the ability of both cell types to adhere to PBS- and fibronectin-coated plastic plates. After 60 min, the number of bound Rab44-KO eosinophils was significantly lower than that of WT eosinophils (**Figure 6A**). Moreover, at a longer incubation period (120 min), similar results were observed in WT and Rab44-KO eosinophils (**Figure 6B**). However, both types of eosinophils showed minimal binding to the control plates (**Figure 6A, B**).

We further analyzed the surface expression of two types of cell-adhesion receptors, integrin β1(CD29) and CD44. Integrin β1 is known to be expressed on the surface of eosinophils and is associated with fibronectins and other matrix proteins, whereas CD44 is a transmembrane glycoprotein that regulates cell-matrix interactions via binding to hyaluronic acid and fibronectin (27, 28). Flow cytometric analysis revealed that the surface expression of integrin β1 was slightly lower in Rab44-KO eosinophils than in the WT cells, although this difference was not statistically significant. (**Figure 6C**). The surface levels of CD44 in Rab44-KO eosinophils were markedly lower than those in WT cells (**Figure 6D**). However, the surface expression of integrin β2 (CD18) in Rab44-KO eosinophils was comparable to that in WT cells (data not shown). Taken together, the reduced cell adhesion ability of Rab44-KO eosinophils is likely due to decreased surface levels of their cell-adhesion receptors.

### 3.7 Rab44-KO eosinophils display decreased chemotaxis and reduced surface levels of chemokine receptors

**Figure 7:**
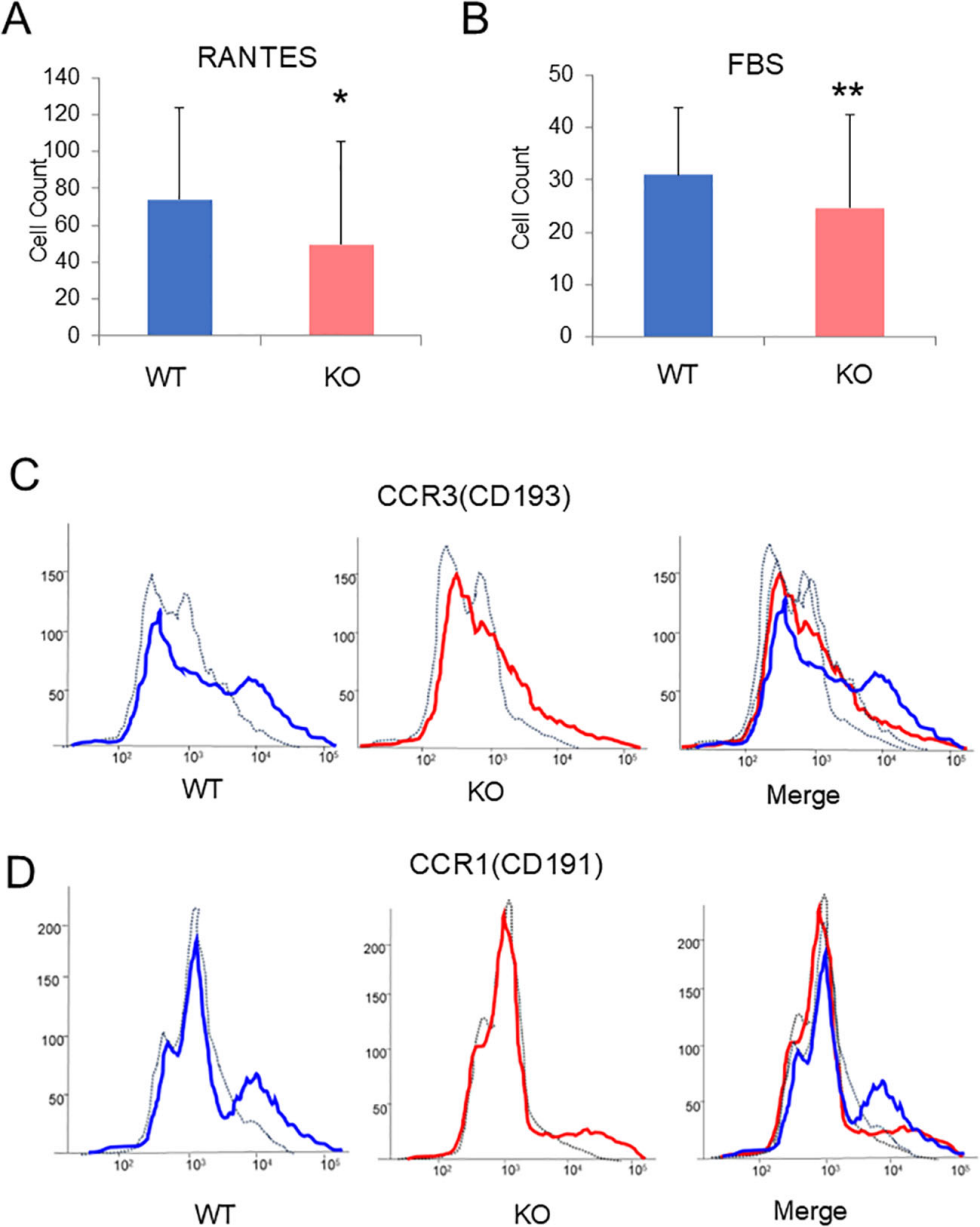
Chemotaxis and surface levels of chemokine receptors in bone marrow eosinophils. Bone marrow eosinophils were washed and resuspended in IMDM without FBS as the control medium. **(A, B)** Migration of cells in response to 10% FBS or 200 ng/mL RANTES was assessed in a 24-well Transwell chemotaxis chamber. The chambers were incubated at 37 °C for 120 min. Cells that migrated to the lower well were fixed with 4% paraformaldehyde, stained with 5% Coomassie Brilliant Blue, and counted using a light microscope. The data are means ± SD for four independent experiments. WT: n = 4; Rab44-KO: n = 4 (samples of eosinophils) * *p < 0.05*, ** *p < 0.01.* **(C, D)** The surface of bone marrow eosinophils (1 × 10^5^ cells) was stained with FITC-labeled antibodies for CCR3 (CD193) **(C)** or CCR1 (CD191) **(D)** and analyzed using flow cytometry. Data are representative of five independent experiments. The Mann-Whitney U test was used.

To examine chemotaxis and chemokine receptors in eosinophils, we analyzed the chemotactic responses of eosinophils in WT and Rab44-KO mice. In response to RANTES, the number of Rab44-KO eosinophils migrating from the upper to the lower chamber was significantly lower than that of WT eosinophils (**Figure 7A**). Similar results were observed for WT and Rab44-KO eosinophils in experiments with additional FBS, although the number of migrating cells was decreased (**Figure 7B**).

To evaluate the cell surface levels of chemokine receptors, we examined the surface expression of CCR3 (CD193), a major eosinophil chemokine receptor, and CCR1 (CD191), a chemokine receptor for several ligands including CCL3 and CCL5, using flow cytometry. As shown in **Figure 7C, D**, Rab44-KO eosinophils exhibited significantly lower levels of both CCR3 and CCR1 than WT cells. These results indicate that the decreased chemotaxis in Rab44-KO eosinophils is probably at least partly due to low surface levels of chemokine receptors.

## 4 Discussion

In this study, we demonstrated that Rab44 deficiency impairs OVA-induced allergic airway inflammation. Histopathological analyses revealed that, compared with WT mice, Rab44-KO mice showed decreased inflammatory reactions in the peribronchial and perivascular areas of the lungs under following OVA induction. RNA-seq analysis indicated that Rab44 deficiency affected several signaling pathways involved in immune and inflammatory responses in the lungs of OVA-induced mice. Biochemical analysis showed that Rab44-KO mice displayed reduced immune responses and decreased eosinophil infiltration in BALF under OVA-induced conditions. In *in vitro* experiments with cultured eosinophils, Rab44-KO eosinophils exhibited aberrant differentiation and impaired release of EPX but not MBP. Rab44-KO eosinophils exhibited decreased cell adhesion and chemotaxis. Consistent with these findings, Rab44-KO eosinophils showed reduced levels of surface adhesion and chemokine receptor levels. Therefore, Rab44 deficiency may impair the development of OVA-induced allergic airway inflammation by modulating immune responses and eosinophil functions.

The finding that Rab44 deficiency reduces immune responses in allergic airway inflammation is consistent with previous *in vivo* studies showing that Rab44-KO mice exhibited decreased allergic responses, including anaphylaxis and nickel-induced hypersensitivity (18, 19). Transcriptomic analysis of patients with atopy and atopic asthma revealed that the expression levels of Rab44 in these patients were higher than those in healthy individuals (29). Moreover, a genetic study of autoimmune diseases showed that a missense mutation in *Rab44* has been identified in patients with CD4-lymphoproliferative disease (30). Taken together, Rab44 deficiency appears to impair immune responses, regardless of allergic or autoimmune disease status.

RNA-seq analysis indicated that Rab44 deficiency affected several signaling pathways involved in immune and inflammatory responses in the lungs of OVA-induced mice. However, these changes likely reflect secondary consequences of attenuated inflammation in Rab44-KO mice, rather than direct mechanistic effects To further delineate Rab44-specific functions, we focused on the gene sets associated with cellular trafficking and immune cell migration. Notably, several genes involved in receptor internalization and chemokine signaling were differentially expressed under OVA-induced conditions. Given that Rab proteins are key regulators of intracellular trafficking, these transcriptional changes suggest that Rab44 deficiency may impair the proper localization or recycling of surface receptors required for eosinophil function. This interpretation is consistent with our hypothesis that Rab44 regulates eosinophil trafficking. The altered expression of chemokine-related genes may influence CCR3-mediated signaling and downstream migratory responses, thereby contributing to the reduced eosinophil accumulation observed in the airway. Moreover, the involvement of immunoglobulin variable genes and viral interaction pathways may have secondary effects on B cells and other immune-related cells. Thus, rather than representing generic inflammatory signatures, the RNA-seq data provide indirect evidence that Rab44 modulates immune cell dynamics through trafficking-dependent mechanisms.

Experiments examining the expression levels of eosinophil markers demonstrated that Rab44-KO eosinophils exhibited aberrant differentiation, characterized by decreased mRNA expression of IL-5Rα, CCR3, and CD11b, and increased expression of CD62L. Since CD62L serves as a marker for eosinophil subpopulations, with CD62L-high identifying resident eosinophils (rEos) and CD62L-low or -negative identifying inflammatory eosinophils (iEos) (31), these data suggest that Rab44-KO eosinophils may display a more resident-like and less inflammatory phenotype compared to WT cells. Thus, these findings raise the possibility that Rab44 influences eosinophil differentiation rather than causing intrinsic defects in mature eosinophils. We have previously shown that Rab44 is involved in the differentiation of osteoclasts and myoblasts (11, 14, 15). In osteoclast differentiation, Rab44 regulates lysosomal pH and Ca^2+^ influx (11), whereas in myoblast differentiation, Rab44 modulates mTORC1 signaling and the cell surface transport of fusion-related proteins (14, 15). Based on these findings, it will be important to elucidate the molecular mechanisms underlying eosinophil differentiation mediated by Rab44.

Regarding secretion experiments, Rab44 deficiency affects the release of EPX, but not MBP. Previous morphological studies have shown that EPX localizes predominantly within the surrounding granule matrix, whereas MBP is primarily distributed within the crystalloid core of eosinophil granules (32–34), suggesting differential secretion of EPX and MBP. Eosinophils exhibit three main modes of degranulation: piecemeal degranulation, classical exocytosis, and cytolysis (4, 35). Piecemeal degranulation involves selective release via vesicular transport in which EPX is preferentially released, whereas MBP is released less readily (36, 37). In contrast, classical exocytosis and cytolysis release both EPX and MBP, respectively. Thus, Rab44 may be involved in piecemeal degranulation. Interestingly, human Rab44 in eosinophils has been reported to localize in crystalloid granules in both unstimulated and IL-33-stimulated cells and translocate to the nucleopod after stimulation with IL-5 (38). Furthermore, a previous phospho-proteomic analysis indicated that Rab44 was highly phosphorylated upon prolonged stimulation (20 h) with IL-3 (39) or brief stimulation (10 min) with IL-33 (38), whereas it showed comparatively lower levels of phosphorylation after acute stimulation (5 min) with IL-5 (40). Therefore, our findings may contribute to understanding of eosinophil degranulation regulated by Rab44.

Moreover, our findings indicated that Rab44-KO eosinophils showed reduced cell adhesion and chemotaxis. To date, research on the regulation of eosinophil adhesion receptors and chemokine receptors by Rab proteins remains limited (41). Studies on the involvement of Rab proteins in eosinophils have primarily focused only on degranulation mediated by Rab27A. Peripheral blood eosinophils derived from Rab27A mutant *Ashen* mice exhibited defective EPX secretion both *in vitro* and *in vivo* (42, 43). Because Rab27A is involved in receptor trafficking in other immune-related cells (44, 45), it may also be responsible for receptor trafficking in eosinophils, similar to Rab44. Taken together, these findings suggest that Rab44 regulates the expression of chemokines and integrin receptors, thereby decreasing chemotaxis and cell adhesion ability.

In summary, Rab44-KO mice exhibit reduced inflammatory reactions in the peribronchial and perivascular areas of the lungs under OVA-induced conditions. Consistent with these findings, RNA-seq analysis indicated that Rab44 deficiency affected several signaling pathways involved in the immune and inflammatory responses in the lungs of OVA-induced mice. Rab44-KO eosinophils exhibit reduced cell adhesion and chemotactic abilities, with decreased surface levels of their receptors. Thus, loss of Rab44 may attenuate OVA-induced allergic airway inflammation by modulating immune responses and eosinophil function.

### 4.1 Limitations of the study

In the present study, we conducted *in vivo* experiments using Rab44-KO and WT mice sensitized with OVA to investigate allergic airway inflammation. In addition, we performed *in vitro* experiments using cultured eosinophils. However, our study has several limitations. First, our experiments were primarily conducted using a murine model. Further studies are required to confirm whether similar mechanisms are observed in human patients or cells. Second, total lung RNA was analyzed, not sorted or single cells in the RNA-seq analysis. Third, as the *in vitro* analysis of asthma-related immune cells was limited to eosinophils, it is necessary to include analyses of other immune cell populations. Finally, changes in gene expression in the lungs were analyzed at the transcript level; however, protein-level validation was not fully conducted.

## Data Availability

The original contributions presented in the study are publicly available. This data can be found here: NCBI SRA/Bioproject, accession PRJNA1466819 and NCBI GEO, accession GSE331548.
